# Comparing Adherence to the Experience Sampling Method Among Patients With Schizophrenia Spectrum Disorder and Unaffected Individuals: Observational Study From the Multicentric DiAPAson Project

**DOI:** 10.2196/42093

**Published:** 2023-07-18

**Authors:** Cristina Zarbo, Manuel Zamparini, Olav Nielssen, Letizia Casiraghi, Matteo Rocchetti, Fabrizio Starace, Giovanni de Girolamo, DiAPAson Collaborators

**Affiliations:** 1 Unit of Epidemiological and Evaluation Psychiatry IRCCS Istituto Centro San Giovanni di Dio Fatebenefratelli Brescia Italy; 2 Department of Psychology University of Milano Bicocca Milano Italy; 3 MindSpot Clinic Macquarie University Sydney Australia; 4 School of Psychological Sciences Faculty of Medicine, Health and Human Sciences Macquarie University Sydney Australia; 5 Department of Brain and Behavioural Sciences University of Pavia Pavia Italy; 6 Department of Mental Health and Dependence ASST of Pavia Pavia Italy; 7 Department of Mental Health and Dependence AUSL of Modena Modena Italy; 8 see Acknowledgments

**Keywords:** ecological momentary assessment, multicenter study, mobile application, mobile app, compliance, psychosis

## Abstract

**Background:**

The Experience Sampling Method (ESM) is a valid method of remotely recording activities and mood, but the predictors of adherence to ESM in patients with Schizophrenia Spectrum Disorder (SSD) are not known. Studies on adherence are significant as they highlight the strengths and weaknesses of ESM-based study designs and allow the development of recommendations and practical guidelines for implementing future studies or treatment plans.

**Objective:**

The aim of this study was to compare the adherence to ESM in patients with SSD and unaffected control individuals, investigate their patterns, and report the predictors of adherence.

**Methods:**

In total, 131 patients with SSD (74 in residential facilities and 57 outpatients) and 115 unaffected control individuals were recruited at 10 different centers in Italy as part of the DiAPAson project. Demographic information, symptom severity, disability level, and level of function were recorded for the clinical sample. Participants were evaluated for daily time use and mood through a smartphone-based ESM 8 times a day for 7 consecutive days. Adherence was measured by the response rate to ESM notifications. Results were analyzed using the chi-square test, ANOVA, Kruskal-Wallis test, and Friedman test, and a logistic regression model.

**Results:**

The overall adherence rate in this study was 50% for residents, 59% for outpatients, and 78% for unaffected control individuals. Indeed, patients with SSD had a lower rate of adherence to ESM than the unaffected control group (*P≤*.001), independent of time slot, day of monitoring, or day of the week. No differences in adherence rates between weekdays and weekends were found among the 3 groups. The adherence rate was the lowest in the late evening time slot (8 PM to 12 AM) and days 6-7 of the study for both patients with SSD and unaffected control individuals. The adherence rate among patients with SSD was not predicted by sociodemographic characteristics, cognitive function, or other clinical features. A higher adherence rate (ie, ≥70%) among patients with SSD was predicted by higher collaboration skills (odds ratio [OR] 2.952; *P=*.046) and self-esteem (OR 3.394; *P=*.03), and lower positive symptom severity (OR 0.835; *P=*.04).

**Conclusions:**

Adherence to ESM prompts for both patients with SSD and unaffected control individuals decreased during late evening and after 6 days of monitoring. Higher self-esteem and collaboration skills predicted higher adherence to ESM among patients with SSD, while higher positive symptom scores predicted lower adherence rates. This study provides important information to guide protocols for future studies using ESM. Future clinical or research studies should set ESM monitoring to waking hours, limit the number of days of monitoring, select patients with more collaborative skills and avoid those with marked positive symptoms, provide intensive training sessions, and improve participants’ self-confidence with technologies.

**International Registered Report Identifier (IRRID):**

RR2-10.1186/s12888-020-02588-y

## Introduction

The Experience Sampling Method (ESM) has gained wide acceptance as a tool for the measurement of a range of psychological, psychophysiological, cognitive, and behavioral data in real-life settings among different populations [1-3], including patients with a diagnosis of Schizophrenia Spectrum Disorders (SSD) or psychosis [4-16]. The integration of ESM in research studies and clinical practice helps limit potential recall bias and the impact of decreased cognitive capacities, providing longitudinal ecological data and capturing the variability over time and dynamic patterns of reactivity to the context [2,17]. Moreover, an ecological understanding of these patients is important for assessing the level of disability or the effect of treatments and rehabilitation programs.

Despite it being a widespread practice among mental health researchers, few studies focused on the evaluation of adherence (ie, the degree to which a person’s behavior coincides with the given instructions) to the ESM protocol among patients with SSD [6,8,10-13,15,18,19], reporting mixed results. Indeed, the adherence rates reported in previous studies among patients with an SSD range from about 30% [18] to 87% [6].

Furthermore, relatively little is known about the factors associated with adherence to ESM among patients with severe mental disorders, although previous studies reported that some individual, contextual, or method-based factors may have a significant role. For example, some studies found that recent cannabis use [18], higher symptom severity or worse premorbid adjustment [16,20], lower executive functioning [16], a higher number of lifetime suicide attempts [20], lower psychosocial function [10], male gender [21], older age [16], lower educational level [16], the timing of prompts [11,22], a higher number of assessments per day [21], and lower incentives [21] predicted lower adherence to ESM. A recent mixed methods study [19] found that living in residential facilities (RF) was associated with lower adherence levels, and that residents with SSD report more problems related to the use of smartphones often display feelings of inadequacy and low self-confidence and also exhibit difficulties responding to notifications at certain times, probably due to both the routines of RFs and the effect of negative symptoms.

In order to validate the results of ESM-based studies in patients with SSD and establish the predictors of adherence, a study comparing the results of an ESM-based evaluation among patients with SSD with unaffected control individuals was conducted as part of a large multicenter study assessing the day-to-day activities of patients with SSD. Our aims were to (1) evaluate the rates and patterns of adherence to ESM among patients with SSD in residents, outpatients, and unaffected control individuals and (2) investigate the sociodemographic and clinical predictors of adherence to ESM for both outpatients and residents.

## Methods

### Participants and Procedure

From October 2020 to October 2021, a total of 131 patients with a diagnosis of SSD (74 residents and 57 outpatients) and 115 unaffected controls were recruited at 10 different centers in Northern Italy, as part of the DiAPAson project [19,23-26]. We included patients with an SSD diagnosis based on the Diagnostic and Statistical Manual of Mental Disorders, Fifth Edition (DSM-5) [27], who were 20-55 years old, able to speak and write in Italian, and were undergoing treatment at psychiatric RFs or as outpatients at a department of mental health. We excluded patients who were deemed unable to provide informed consent or who had significant cognitive deficits (ie, a Mini-Mental State Examination corrected score lower than 24), a recent diagnosis of substance use disorder according to DSM-5 criteria [27], a history of clinically significant head injury, or degenerative neurological disease.

Unaffected control individuals were recruited by public advertisements and snowball sampling procedures and were matched by gender and age group (ie, 20-24, 25-29, 30-34, 35-39, 40-44, 45-49, and 50-55 years) with the clinical sample. At each study center, treating clinicians invited participants under their care to participate in the study. Participants were provided with detailed information about the study and had the opportunity to ask questions. Some of the assessment questionnaires were completed by the treating clinician, and research assistants (RA) helped the patients complete self-report questionnaires. ESM monitoring was preceded by a briefing session in which the RA gave instructions about the ESM notifications and how to effectively respond. The monitoring was followed by a debriefing session in which the same RA collected information on study acceptability and feasibility. During the debriefing session, outpatients and unaffected control individuals received €25 (US $27.38) for travel expenses.

### Measures

Participants were assessed with a questionnaire recording psychiatric history and current treatment, an assessment of collaboration skills, the Specific Levels of Functioning Scale (SLOF) [28], the Brief Psychiatric Rating Scale (BPRS) [29,30], the Brief Negative Symptom Scale (BNSS) [31,32], and the World Health Organization Disability Assessment Schedule 2.0 (WHODAS 2.0) [33,34]. Self-esteem was assessed with the question, “*I think I am at least as good as the others*” derived from the Italian version of the Rosenberg Self-Esteem Scale [35].

Daily usage time and mood were measured using a smartphone-based ESM (ie, the participants completed a brief questionnaire about their current activities and mood on a smartphone 8 times a day from 8 AM to 12 AM for 7 consecutive days. Notifications were semirandomized (ie, were randomly sent within 8 scheduled time slots) and a reminder notification was sent after 15 minutes if there was no response to the initial prompt. The participant was allowed a maximum of 30 minutes to answer the notification.

This study was conducted in accordance with American Psychological Association’s [36] ethical standards for the treatment of human experimental volunteers and each participant provided consent in compliance with the tenets of the Declaration of Helsinki [37]. This study was approved by the ethical committee (EC) of the local institutions.

### Ethical Considerations

The study has been approved by the ECs of the 3 main participating centers: EC of IRCCS (Istituto di Ricovero e Cura a Carattere Scientifico) Istituto Centro San Giovanni di Dio Fatebenefratelli (July 31, 2019, number 211/2019), EC of Area Vasta Emilia Nord (September 25, 2019, number 0025975/19), and EC of Pavia (September 2, 2019, number 20190075685) and by the ECs of all participating sites. Data were deidentified in order to prevent the participants’ personal identities from being revealed.

### Statistical Analyses

Kolmogorov-Smirnov tests were performed as preliminary analyses to ensure no violation of the assumption of normality. Adherence with mobile ESM was measured by calculating the reaction rates to notifications (expressed as the percentage of answers), initially over a total of 56 notifications for 7-day monitoring, and then considering separate time slots (8 AM to 12 PM, 12 PM to 4 PM, 4 PM to 8 PM, and 8 PM to 12 AM) and days of the study (1-2, 3-5, and 6-7). No participants were excluded for not having reached a minimum of answered notifications in this study.

The descriptive statistics of sociodemographic and clinical variables consist of frequency tables (for categorical variables) and means and SDs (for continuous variables). To assess differences between groups, we used chi-square tests (or the Fisher exact test, which is more appropriate when smaller numbers are considered) and ANOVA (or the nonparametric Kruskal-Wallis test for variables not normally distributed). Bonferroni correction was applied for post hoc group comparisons.

For aim 1, we computed the percentage of adherence in the 3 different groups and for different daily hours and days, and we checked for any significant differences in rows (between groups) using a Kruskal-Wallis test, and differences in columns (within groups) using the Friedman test. For aim 2, we used binomial logistic regression models (separately for residents and outpatients), considering an adherence rate of ≥70% as the dependent variable to indicate a predictor of adherence. This cut-off was established in accordance with previous ESM studies that considered at least about 30% of answered notifications as acceptable [8,38].

All statistical analyses were performed using SPSS (version 27.0; IBM Corp). All statistical tests were 2-tailed; a *P* value of ≤.05 was considered significant.

## Results

### Sociodemographic and Clinical Characteristics of the Sample

The 3 groups were similar in their age and gender distribution (Table 1). Patients had lower levels of education and employment than control individuals, and the residents had more severe conditions: a longer illness duration (mean 19.3, SD 10.0 years), higher rates of psychiatric comorbidities (n=31, 41.9%), higher numbers of both antipsychotic (mean 1.7, SD 0.7) and nonantipsychotic medications (mean 1.6, SD 1.2), greater symptom severity (mean total BPRS score 47.0, SD 13.2), more severe negative symptoms (mean BNSS score 23.5, SD 14.6), and lower levels of social function (mean SLOF score 175.8, SD 20.7).

**Table 1:** Sociodemographic and clinical characteristics of the study sample.

Characteristics		Residents (n=74)		Outpatients (n=57)		Unaffected control individuals (n=115)		*P* value	
Age (years), mean (SD)		42.8 (10.4)		38.6 (10.7)		41.6 (10.2)		.06	
Gender (male), n (%)		52 (70.3)		32 (56.1)		69 (60.0)		.20	
Education (years), mean (SD)^a^		11.4 (3.5)		12.5 (2.5)		16.6 (4.9)		<.001	
Working or studying (yes), n (%)		16 (21.6)		35 (61.4)		113 (99.1)		<.001	
MMSE^b^ score, mean (SD)		27.3 (1.5)		27.7 (1.1)		N/A^c^		.33	
Illness duration (years), mean (SD)		19.3 (10.0)		15.6 (8.9)		N/A		.04	
Psychiatric comorbidities (yes), n (%)		31 (41.9%)		12 (21.1%)		N/A		.01	
**Collaboration skills, n (%)**									.26
	High collaboration skills	35 (48.0)	33 (57.9)		N/A				
	Low-medium collaboration skills	38 (52.0)	24 (42.1)		N/A				
Number of antipsychotic medications, mean (SD)		1.7 (0.7)		1.4 (0.8)		N/A		<.001	
Number of nonantipsychotic medications, mean (SD)		1.6 (1.2)		0.9 (0.8)		N/A		<.001	
**Self-esteem, n (%)**									.29
	High self-esteem	27 (38.0)	27 (47.4)		N/A				
	Low-medium self-esteem	44 (62.0)	30 (52.6)		N/A				
**Total BPRS^d^ score, mean (SD)**		47.0 (13.2)		41.2 (9.8)		N/A		.02	
	Psychotic symptoms	9.2 (4.1)	8.1 (3.8)		N/A		.13		
	Depression or anxiety	14.7 (5.3)	13.9 (4.1)		N/A		.66		
	Manic excitement	10.2 (4.9)	8.4 (3.2)		N/A		.049		
	Cognitive symptoms	4.8 (2.0)	3.8 (1.1)		N/A		.004		
	Negative symptoms	8.2 (3.4)	6.9 (2.9)		N/A		.02		
Total BNSS^e^ score, mean (SD)		23.5 (14.6)		17.1 (13.4)		N/A		.01	
Total SLOF^f^ score, mean (SD)		175.8 (20.7)		191.4 (15.2)		N/A		<.001	
Total WHODAS 2.0^g^ score, mean (SD)		10.9 (8.1)		10.3 (7.7)		N/A		.72	

^a^On post hoc analysis, we found that unaffected control individuals had significantly more years of education than residents or outpatients (*P*<.001).

^b^MMSE: Mini-Mental State Examination.

^c^N/A: not applicable.

^d^BPRS: Brief Psychiatric Rating Scale.

^e^BNSS: Brief Negative Symptom Scale.

^f^SLOF: Specific Levels of Functioning Scale.

^g^WHODAS 2.0: World Health Organization Disability Assessment Schedule 2.0.

### Rates of Adherence to the ESM

**Figure 1:**
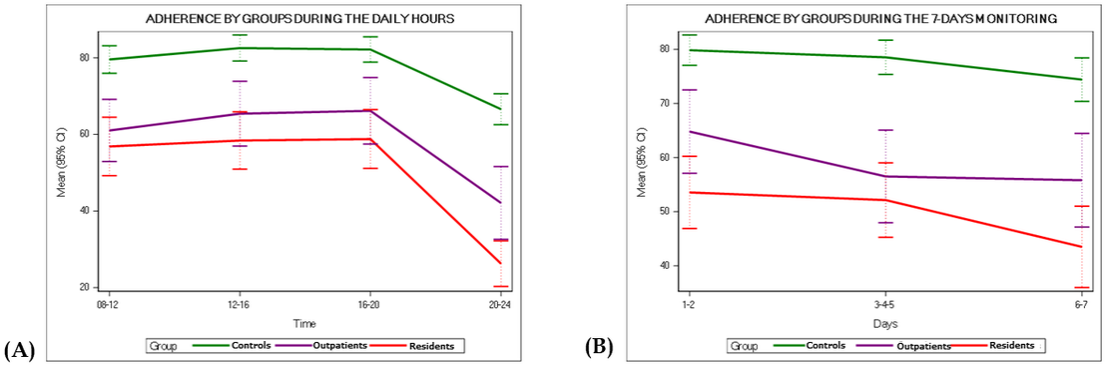
Adherence rate by groups (A) during daily hours and (B) throughout the 7-day monitoring period.

Between-group analyses (Table 2 and Figure 1) revealed that the number of participants to be excluded due to <30% of ESM notifications being answered was significantly higher among residents (n=17, 23%) than among outpatients (n=10, 17.5%) and unaffected control individuals (n=3, 2.6%). Moreover, residents had the lowest adherence rate among all 3 groups on weekdays (ie, Monday to Friday; mean 49.4%, SD 27.7%), in the 8 PM to 12 AM time slot (mean 26.3%, SD 25.7%), and in the first 2 (mean 53.5%, SD 28.8%) and the last 2 (mean 43.5%, SD 32.5%) days of monitoring. However, residents and outpatients showed similar adherence rates when considering the total adherence rate on weekends, in the 8 AM to 8 PM time slots, and in the central days of monitoring (ie, days 3, 4, and 5). Control group participants showed the highest adherence rate independent of daily time, day of monitoring, and day of the week.

Within-groups analyses (Table 2 and Figure 1) showed no differences in adherence rates among the 3 groups between weekends and weekdays. Again, not surprisingly, the 8 PM to 12 AM time slot had the lowest adherence rate among the 3 groups. Similarly, adherence rates were the lowest on the last 2 days between residents and control participants.

**Table 2:** Rates of adherence to the Experience Sampling Method (ESM).

		Residents (R)	Outpatients (O)	Unaffected control individuals (C)	*P* value	Post hoc comparisons
<30% responses to ESM notifications (%), n (%)		17 (23.0)	10 (17.5)	3 (2.6)	<.001	N/A^a^
Total adherence during the 7-day study period (%), mean (SD)		50.1 (27.4)	58.7 (29.0)	77.7 (16.1)	<.001	C>R or O^b^
Adherence rate on weekdays (Monday-Friday; %), mean (SD)		49.4 (27.7)	60.3 (27.6)	78.0 (17.1)	<.001	R<O<C
Adherence rate on weekends (Saturday-Sunday; %), mean (SD)		51.7 (29.9)	54.6 (35.6)	77.0 (17.2)	<.001	C>R or O
*P* value (comparing adherence between weekdays and weekends)		.45	.052	.33	N/A	N/A
**Adherence during time slots^c^ (%), mean (SD)**						
	8 AM to 12 PM	56.9 (33.0)	61.0 (30.6)	79.6 (19.5)	<.001	C>R or O
	12 PM to 4 PM	58.4 (32.3)	65.4 (31.9)	82.5 (18.4)	<.001	C>R or O
	4 PM to 8 PM	58.8 (33.1)	66.2 (32.7)	82.2 (17.9)	<.001	C>R or O
	8 PM to 12 AM	26.3 (25.7)	42.1 (35.8)	66.6 (21.8)	<.001	R<O<C
*P* value (comparing adherence among the aforementioned time slots)		<.001	<.001	<.001	N/A	N/A
**Adherence during days of the study^d^ (%), mean (SD)**						
	1-2	53.5 (28.8)	64.8 (29.1)	79.8 (15.2)	<.001	R<O<C
	3-5	52.1 (29.6)	56.5 (32.2)	78.5 (17.1)	<.001	C>R or O
	6-7	43.5 (32.5)	55.8 (32.6)	74.4 (21.8)	<.001	R<O<C
*P* value (comparing adherence among the aforementioned days of the study)		.005	.12	.049	N/A	N/A

^a^N/A: not applicable.

^b^Post hoc comparison revealed that adherence was higher among unaffected control individuals than among residents and outpatients.

^c^Post hoc comparisons revealed that adherence was the lowest in the 8 PM to 12 AM time slot.

^d^Post hoc comparisons revealed that adherence was the lowest on days 6-7 of the study.

### Predictors of Adherence to the ESM

Logistic regression analyses (Table 3) revealed that a higher adherence rate (ie, ≥70%) was significantly predicted by higher collaboration skills (odds ratio [OR] 2.95, 95% CI 1.02-8.54; *P=*.046) among residents. Among outpatients, higher self-esteem (OR 3.39, 95% CI 1.13-10.14; *P=*.03) and lower severity of positive symptoms (OR 0.83, 95% CI 0.70-0.98; *P=*.04) predicted a higher adherence rate (ie, ≥70%).

**Table 3:** Predictors of adherence with the Experience Sampling Method (cutoff=70%).

			Residents				Outpatients		
			Odd ratios (95% CI)		*P* value		Odds ratio (95% CI)		*P* value
Age			1.007 (0.958-1.058)		.79		0.989 (0.941-1.039)		.66
Gender (male)			0.789 (0.266-2.340)		.67		1.324 (0.459-3.818)		.60
Education			1.050 (0.908-1.214)		.51		1.133 (0.907-1.415)		.27
Collaboration skills			2.952 (1.020-8.547)		.046		1.167 (0.403-3.374)		.78
Working or studying (yes)			0.293 (0.60-1.423)		.13		0.492 (0.167-1.455)		.20
Total MMSE^a^ score			0.940 (0.676-1.306)		.71		1.530 (0.904-2.589)		.11
Illness duration			1.020 (0.969-1.074)		.45		1.028 (0.968-1.091)		.37
Psychiatric comorbidities (no)			0.551 (0.199-1.529)		.25		1.120 (0.308-4.067)		.86
Taking antipsychotic medications			0.733 (0.351-1.533)		.41		0.541 (0.251-1.170)		.12
Taking nonantipsychotic medications			1.431 (0.946-2.165)		.09		1.553 (0.804-3.000)		.19
Self-esteem			1.333 (0.472-3.770)		.59		3.394 (1.135-10.145)		.29
**Total BPRS^b^ score**			0.996 (0.958-1.036)		.86		0.971 (0.918-1.027)		.30
	Positive symptoms	1.026 (0.908-1.159)		.68		0.835 (0.705-0.988)		.04	
	Depression and anxiety	1.016 (0.924-1.117)		.75		1.033 (0.909-1.175)		.62	
	Manic excitement	1.004 (0.904-1.114)		.95		0.958 (0.809-1.135)		.62	
	Cognitive symptoms	1.020 (0.792-1.313)		.88		0.636 (0.370-1.094)		.10	
	Negative symptoms	0.847 (0.712-1.007)		.06		0.985 (0.820-1.184)		.88	
Total BNSS^c^ score			0.986 (0.952-1.022)		.46		0.998 (0.960-1.038)		.93
Total SLOF^d^ score			1.013 (0.988-1.039)		.32		1.000 (0.965-1.035)		.98
Total WHODAS 2.0^e^ score			0.978 (0.916-1.043)		.50		0.938 (0.869-1.012)		.10

^a^MMSE: Mini-Mental State Examination.

^b^BPRS: Brief Psychiatric Rating Scale.

^c^BNSS: Brief Negative Symptom Scale.

^d^SLOF: Specific Levels of Functioning Scale.

^e^WHODAS 2.0: World Health Organization Disability Assessment Schedule 2.0.

## Discussion

### Background

In this study, we aimed at assessing the adherence rate to the ESM among patients with SSD (divided into resident and outpatient groups) and unaffected control individuals across daily hours and days of monitoring, and the factors associated with adherence in different patient groups. In the next sections, we discuss the main findings of our study and their implications for planning of real-time research with clinical populations with SSD.

### Principal Findings

#### Patients With SSD Are Poorly Adherent to ESM Compared to Unaffected Control Individuals

Patients with SSD, especially those living in a residential setting, who had more severe illness and greater disability, were less adherent to the ESM than unaffected control individuals (ie, 50%-58% vs 78%), independent of the time or day of monitoring. This result confirms that of previous studies, which found lower adherence to the ESM among patients with SSD than among control participants [10,19,39]. The overall adherence rates of 50% for residents and 58% for outpatients in this study were somewhat lower than those reported by Rintala et al [39] (ie, 70%), Granholm et al [8] (ie, 85%), Granholm et al [6] (ie, 87%), Jones et al [40] (ie, >80%), and So et al [41] (ie, 70.7%), but these rates were higher than those reported by Moitra et al [18] (ie, 28%-31%) among patients recently discharged from hospital. However, due to the specific purpose of this study, it should be highlighted that we did not exclude any participants for low rates of answered notifications, and this may have decreased the overall adherence rate.

Consistent with the experience of other researchers [18,19], we hypothesize that adherence was affected by the general difficulties encountered by patients in their use of smartphones, including impaired problem-solving skills when faced with technical problems. Moreover, many patients with SSD, especially those in residential settings, have some illness- or treatment-related cognitive difficulties, including psychomotor slowing commonly found in these individuals [42], and often do not own their own smartphone and were using a smartphone for the first time. The limited time available to answer each notification may have affected the adherence rate in residential facilities with structured daily activities. Unfortunately, we were unable to collect information about who required support and how much, for completing ESM questionnaires due to individual limitations.

#### Adherence to the ESM Decreases in the Evening and After 5 Days of Monitoring

The adherence to ESM prompts for both patients and unaffected control individuals decreased in the late evenings (ie, in the 8 PM to 12 AM time slot) and after 6 days of monitoring (ie, on days 6-7). The effect of the daily hours on the adherence rate was different from that reported by Rintala et al [39]: the latter found the lowest compliance between 7:30 AM and 9 AM. However, in line with our results, Rintala et al [39] also found that compliance declined across days, being the lowest on the fifth day, whereas Moitra et al [18] found a significant decrease in adherence in the fourth week, admittedly from a lower base rate. On the contrary, Jones et al [40] found that adherence to the ESM was not correlated with the duration of the study.

We may hypothesize that adherence to the ESM was lower in the evenings, especially for residential patients, because of routines in RFs (for instance, going to bed in the early evening) as well as the sedative effect of medication [43]. Indeed, our study design covered all daily hours and was not sensitive to bedtime, afternoon naps, and specific rules on smartphone use and bedtime in the RF. For control participants, the lower adherence rate may be due to not only other activities taking place in the evenings but also going to bed early in order to get up for work the next day. Furthermore, adherence may have declined over the 7 days of monitoring because of the decline in interest in the study after several days. These results suggest that ESM monitoring, especially if considering patients with SSD, may be reliable and feasible within a restricted number of days and daily hours.

#### Do Self-Esteem, Collaboration Skills and Psychiatric Severity Affect ESM Adherence?

Our findings show that higher self-esteem and collaboration skills predicted higher adherence to ESM among patients with SSD, while higher positive symptom scores predicted lower adherence rates. By contrast, we found no effect of sociodemographic characteristics (eg, age, gender, education, and employment status), cognitive function, and other clinical features (eg, illness duration, psychiatric comorbidities, medical treatment, disability level, and level of social function) on adherence. These results differ from those of previous studies, where gender [39], age [39], recent cannabis use [18], and psychosocial function [10] affected ESM adherence among patients with SSD. However, we need to specify that our study excluded a priori patients with recent substance abuse and those with impaired cognitive abilities. Therefore, the lack of significance for these 2 variables may be due to this selection bias. The positive effect of self-esteem on adherence to the ESM is of particular interest, as it has still not been reported, and may indicate a level of self-efficacy or initiative. This result is consistent with those of studies reporting that higher self-esteem is associated with higher adherence to both medical and psychological treatments among patients with SSD [44,45]. It may be assumed that high self-esteem might generate self-confidence, which may, in turn, promote good self-care, including adherence to treatment or active collaboration with interventions promoted by clinical staff, such as ESM monitoring.

By contrast, higher positive symptoms scores, which can include the symptoms of suspiciousness, hallucinations, and unusual content of thought, were associated with lower adherence to ESM monitoring. Patients with those symptoms may be more likely to develop suspicious thoughts about the devices themselves or the purpose of monitoring, and this, in turn, may impair their adherence to ESM notifications. Suspicion in technological devices has been reported in previous studies. For example, a study [46] found that 65% of patients with persecutory delusions showed persecutory thinking about computerized characters, while our own study [19] found that some participants with SSD reported persecutory thinking (ie, “Something that follows me everywhere and alerts me”) and delusions about the use of accelerometer biosensors (ie, “It talked to me”).

### Strengths and Limitations

This study has a number of limitations. First, participants had different levels of experience in the use of smartphones, and residents were often helped by health care workers, while outpatients were not; due to the high complexity of the study design and the number of RFs involved in the study, we were unable to assess the adherence rate in relation to the frequency and magnitude of support received by the staff. Second, residents did not receive financial reimbursement, unlike the outpatients and control participants, which may have affected their adherence to the procedure. Third, our findings may not be generalized to older individuals and patients with severe cognitive, motor, or visual deficits or those with substance use disorders. Fourth, our study was of comparatively short duration. Fifth, the study design was not sensitive to bedtime, afternoon naps, and specific rules on smartphone use and bedtime in the RFs. Sixth, we could not evaluate the validity of the answers given to notifications. However, the study has a number of strengths, including the very comprehensive clinical assessment, the relatively large sample size, and multicenter and controlled design.

### Conclusions

This study adds to existing knowledge on the use of the ESM to measure fine-grained activities of patients with SSD in both residential and outpatient settings, and how the adherence of patients with SSD compares to that of unaffected control individuals. Such studies are relevant as they highlight the strengths and weaknesses of ESM’s study designs and allow for the development of recommendations and practical guidelines for implementing future studies or treatment plans that would optimize adherence rates and be acceptable for this specific population. Higher adherence to ESM assessments will, in turn, allow for the collection of reliable and accurate information about the daily experience of such clinical populations and the development of more specific and valid treatment plans for covering their needs.

Based on our results, the recommendations for both researchers and clinicians using the ESM include (1) setting ESM monitoring to waking hours, between 8 AM and 8 PM, and being sensitive to RFs’ rules and restraints, bedtime schedules, and habits of such populations; (2) beginning with several days of familiarization for patient groups and providing more training sessions prior to the start of the monitoring, which also include health care staff and caregivers; (3) limiting the number of days of monitoring; (4) selecting patients with more collaborative skills and excluding those with marked positive symptoms; and (5) as part of the preparation, attempting to improve participants’ self-confidence in the use of the technologies, guaranteeing adequate training and support before the start of the monitoring process. Finally, a major unanswered core issue in ESM research with this population is whether patients with SSD can provide reliable, accurate, and valid self-reports. Future studies should develop and apply accurate methods for assessing the validity of ESM answers in this population.

## Abbreviations

BNSS: Brief Negative Symptom Scale
BPRS: Brief Psychiatric Rating Scale
DSM-5: Diagnostic and Statistical Manual of Mental Disorders, Fifth Edition
EC: ethical committee
ESM: Experience Sampling Method
IRCCS: Istituto di Ricovero e Cura a Carattere Scientifico
OR: odds ratio
RA: research assistant
RF: residential facility
SLOF: Specific Levels of Functioning Scale
SSD: Schizophrenia Spectrum Disorder
WHODAS 2.0: World Health Organization Disability Assessment Schedule 2.0

## Data Availability

The data set used int his study is published with restricted access on the Zenodo platform [48].
